# The Effect of Local and Global Interventions on Epidemic Spreading

**DOI:** 10.3390/ijerph182312627

**Published:** 2021-11-30

**Authors:** Jiarui Fan, Haifeng Du, Yang Wang, Xiaochen He

**Affiliations:** School of Public Policy and Administration, Xi’an Jiaotong University, Xi’an 710049, China; xuanwoxingyun@163.com (J.F.); hexiaochen121vip@163.com (X.H.)

**Keywords:** epidemic, SEIR model, global/local interventions

## Abstract

Epidemic spreading causes severe challenges to the global public health system, and global and local interventions are considered an effective way to contain such spreading, including school closures (local), border control (global), etc. However, there is little study on comparing the efficiency of global and local interventions on epidemic spreading. Here, we develop a new model based on the Susceptible-Exposed-Infectious-Recovered (SEIR) model with an additional compartment called “quarantine status”. We simulate various kinds of outbreaks and interventions. Firstly, we predict, consistent with previous studies, interventions reduce epidemic spreading to 16% of its normal level. Moreover, we compare the effect of global and local interventions and find that local interventions are more effective than global ones. We then study the relationships between incubation period and interventions, finding that early implementation of rigorous intervention significantly reduced the scale of the epidemic. Strikingly, we suggest a Pareto optimal in the intervention when resources were limited. Finally, we show that combining global and local interventions is the most effective way to contain the pandemic spreading if initially infected individuals are concentrated in localized regions. Our work deepens our understandings of the role of interventions on the pandemic, and informs an actionable strategy to contain it.

## 1. Introduction

A pandemic, such as the Spanish Flu, the Ebola virus, and COVID-19, has tremendous impact on human economic productions and life. In early days of the rapid pandemic spreading, especially faced with a lack of vaccines, many countries took global and local interventions, which are essential components of public health. These strategies include travel restrictions, school/workplace closure, border control, cancellation of massive gathering activities, quarantine of exposed individuals, contact tracing and others [1,2,3]. Up to now, more than 150 countries have imposed restrictions on ships/flights/trains, restricting gathering activities, recommending people reduce travel and implementing other restrictions due to COVID-19. These strategies aim to reduce peak size of the pandemic and delay the transmission time, albeit at a large economic and social cost [4,5,6,7]. However, many governments recently propose to reopen the economy, with possibilities of the wide spread of the virus [8,9,10]. The question remains, is it a good idea to lease the global and local interventions? To what extent do these strategies reduce the spreading? Is there a difference in the interventions of infectious diseases with different incubation periods?

Prior studies used mathematical models to study possible underlying mechanisms [11,12,13,14,15,16,17,18,19,20,21,22] and characteristics [23,24,25,26,27] of the epidemic’s spreading. Empirical results also show that interventions reduce the risk of transmission [28,29,30,31,32,33,34,35,36]. For example, initial analyses find that the implementation of the lockdown policy, social distancing and border controls have a restraining effect on the spread of the epidemic [6,31,37,38,39,40,41,42,43]. Prior work also suggests that travel restrictions substantially delay epidemic spread, such as COVID-19, by an average of 3–5 days [31,32]. Given the consequential nature of such spreading, global interventions that aim to reduce travel flows among various regions and local interventions such as school closure and cancelling large gatherings have to be imposed together. However, we still lack of an understanding of which strategy is more effective.

## 2. Materials and Methods

To quantitatively answer this question, we begin with the traditional SEIR model [44,45] since viruses have the characteristics of incubation period, susceptible individuals may become infected by contact with the source of infection *I*-infected, and thus become asymptomatic *E*-exposed state [46]. In the case of COVID-19, the fact that WHO suggested to put exposed individuals in quarantine prompts us to add another compartment *Q* to the traditional SEIR model, describing the quarantine status of exposed individuals [47,48]. To model the global and local interventions, we consider our social system as a modular network with each region as a community, and the network contains both inter- and intra-community interactions.

**Figure 1:**
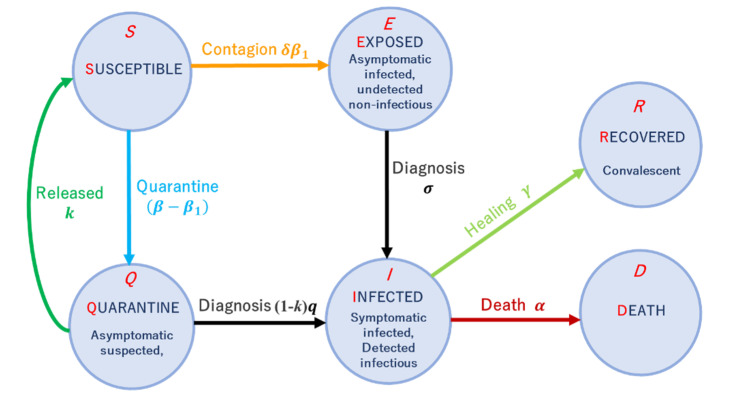
The SQEIR model. Graphical scheme representing the interactions among different stages of infection in the multi-regional-SQEIR model: *S*, susceptible (uninfected); *Q*, quarantine (suspected patients in contact with the infected, undetected); *E*, exposed (infected but did not develop clinical symptoms, undetected, non-infectious); *I*, infected (infected with symptoms, detected, infectious); *R*, recovered (recovered); *D*, death (dead).

We illustrate the model in Figure 1. There are six states in the model: *S*, susceptible (uninfected but can be infected); *Q*, quarantine (suspected patients in contact with the infected, undetected); *E*, exposed (infected but did not develop clinical symptoms, undetected, non-infectious); *I*, infected (infected with symptoms, detected, infectious); *R*, recovered; *D*, deceased.

The spreading dynamics are governed by differential equations as follows:

(1) $$\{\begin{array}{l} \dot{S}=-\left[\delta \beta_{1}+\left(\beta -\beta_{1}\right)\right]SI+kQ\\ \dot{Q}=\left(\beta -\beta_{1}\right)SI-kQ-\left(1-k\right)qQ\\ \dot{E}=\delta \beta_{1}SI-\sigma E\\ \dot{I}=\sigma E+\left(1-k\right)qQ-\left(\gamma +\alpha \right)I\\ \dot{R}=\gamma I\\ S\left(t\right)+Q\left(t\right)+E\left(t\right)+I\left(t\right)+R\left(t\right)+D\left(t\right)=1 \end{array}$$

where $\beta_{1}$, β, σ, and γ quantify the transmission rate to the susceptible status, contact rate with the infected status, diagnosis rate, and recovery rate, respectively. Additionally, *k* represents the fraction of people whose quarantine period was longer than incubation period in the quarantine compartment; *q* represents the rate of diagnosis during quarantine; $\delta =\left(1-\varphi_{intra})⋁(1-\varphi_{inter}\right)$ represents global and local interventions with the OR operator ⋁; $\varphi_{intra}$ and $\varphi_{inter}$ are the strength of local and global interventions, respectively. $\delta =1-\varphi_{intra}$ if node *i* and node *j* are in the same region and local interventions are imposed, $\delta =1-\varphi_{inter}$ otherwise.

In this paper, we study the epidemic spreading on undirected and unweighted networks, denoted by *G*(*V*, *E*), where *V* and *E* represent the set of nodes and edges, respectively [49,50,51,52]. To model the community structure, we use the traditional LFR benchmark network [53], which explains the real-world heterogeneity of node degree and community size. The main parameters of LFR benchmark network are as shown in Table 1.

**Figure 2:**
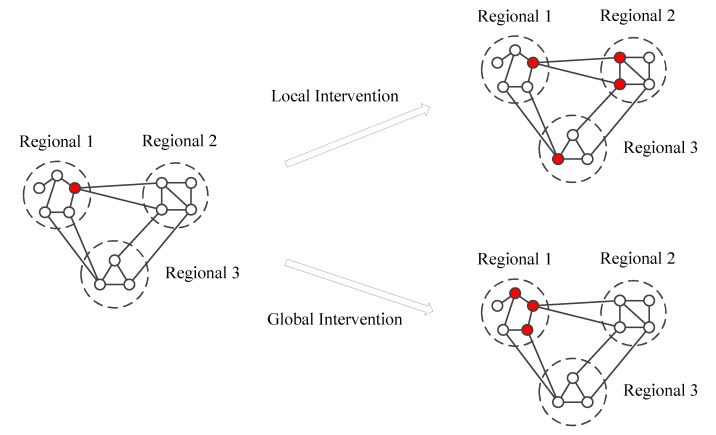
The schematic of the travel restriction of multi-regional-SQEIR model. The solid lines represent relationships between nodes. The dashed-dotted lines represent the regions. The red nodes represent the infected while the white nodes represent the susceptible.

We illustrate global and local interventions in Figure 2. Specifically, the disease easily spreads throughout the whole network without any intervention. With local interventions only such as school closure or local travel restrictions, the disease only spreads among different regions but not within individual regions. Global interventions, on the other hand, efficiently prevents the epidemic spreading among regions.

## 3. Results

### 3.1. The Effectiveness of Global and Local Interventions

**Figure 3:**
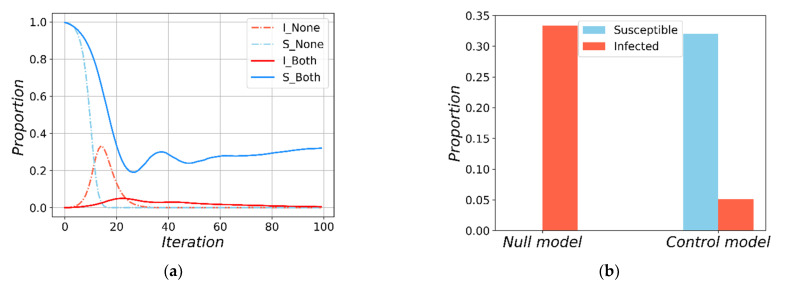
The results of the susceptible and the infected under no travel restrictions and under both local and global restrictions in the multi-regional-SQEIR model. In (**a**), the dashed-dotted lines represent simulations results with no travel restriction, the solid lines represent with both travel restrictions, the red lines represent the fraction of infected individuals, the blue lines represent the fraction of susceptible individuals. In (**b**), the steady state results of the susceptible and the peak results of the infected, the red bar represent the peak of infected individuals, the blue bar represent the steady state of susceptible individuals. (**a**) The transmission processes. (**b**) The steady and peak results.

The first question we ask is, are global and local interventions effective in inhibiting the spread of virus? To this end, we compare the epidemic spreading under the conditions of no travel restriction (the Null model) with the one under both global and local interventions. We set the initial fraction of infected individuals be ρ=0.001 (I(0)≈2). The network initialization and experimental details are in the Supplementary Materials. We find in Figure 3 that implementing global and local interventions has remarkable effects. Specifically, by imposing strict local and global travel restrictions, the proportion of susceptible individuals has a slower decay rate, and the peak of the infected individuals are substantially lower than the Null model. Note that the transmission process of susceptible individuals under travel restrictions show multiple peaks due to the quarantine compartment *Q*, which is consistent with the current real-world scenarios [43,51,54]. In addition, the withdrawal of the peak of the infected under full interventions is also consistent with prior studies [31,32]. Finally, statistical analysis shows that imposing global and local interventions can significantly reduce the peak of infected individuals (Figure 3b, *p*-value = 0.041) by less than one sixth of Null model, and increase the fraction of susceptible individuals (Figure 3b, *p*-value < 10^−6^) by more than 30%. The effectiveness of such interventions prompts us to ask a further question: Are local interventions more effective than global ones, or the other way round?

### 3.2. The Global and Local Interventions

**Figure 4:**
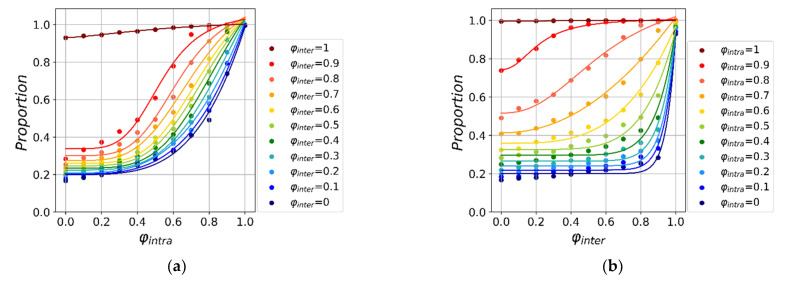
The effect of local and global interventions on epidemic spreading. Here, we vary the intensity of local $\varphi_{intra}$ and global $\varphi_{inter}$ interventions under $\tau_{intra}=\tau_{inter}=0$. The dots in (**a**,**b**) represent the final value of *S* for the given, and the curves of the same color are the nonlinear fitting curves based on the results. (**a**) Fix $\varphi_{intra}$ and adjust $\varphi_{inter}$. (**b**) Fix $\varphi_{inter}$ and adjust $\varphi_{intra}$.

To answer this question, we study the effect of intensity φ and starting time τ of global and local interventions on epidemic spreading. Figure 4 shows the fraction of susceptible individuals when the simulation reaches the steady state. First, we find the proportion of susceptible individual increases as the intervention intensity, demonstrating the effectiveness of such strategy. By comparing Figure 4a with Figure 4b, we find the change rate of the susceptible individual fraction as a function of global and local intervention intensity show substantially different patterns. Specifically, when the local intervention intensity increases, the proportion of susceptible individuals gradually increases from 0.2 to 1, while the same quantity shows abrupt increase as a function of global intervention intensity. In order to carry out further quantitative analysis, we perform a nonlinear fit using a function form as follows:

(2) $$y=\frac{C_{1}}{1+{\left(x/x_{0}\right)}^{a}}+C_{2},$$

where the parameter a represents the tendency of the dependent variable to change. We find the average effect of local intervention intensity is a = 8.00 (Figure 4a), which is substantially larger than global intervention intensity (a = 3.93, Figure 4b). The result shows that the proportion of susceptible individuals changes faster under local interventions, which indicates that local interventions are more effective. Finally, to further quantify the average effect of the different travel restrictions intensities, we use a linear regression, finding the average slope in Figure 4a,b is 0.07 and 0.04, respectively. Specifically, when local intervention intensity increases by 10%, the final survival proportion increases by 7%. On the other hand, the same quantity only increases by 4% when global intervention intensity increases by 10%. Note that strong global interventions are effective only if there is little local intervention.

**Figure 5:**
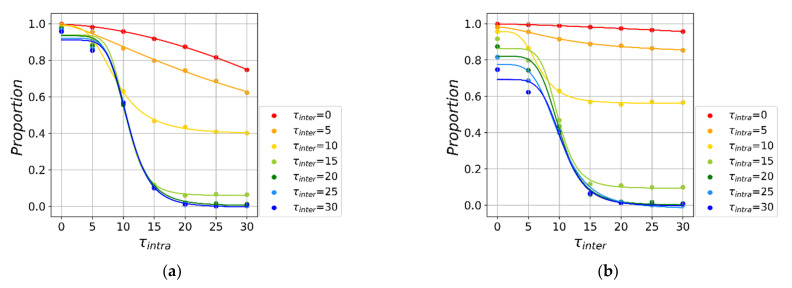
The effect of start time on epidemic spreading. We vary the intensity of local $\tau_{intra}$ and global $\tau_{inter}$ interventions under $\varphi_{intra}=\varphi_{inter}=0.9$. The dots in (**a**,**b**) represent the final value of *S* for the given, and the curves of the same color are the nonlinear fitting curves based on the results. (**a**) Fix $\tau_{intra}$ and adjust $\tau_{inter}$. (**b**) Fix $\tau_{inter}$ and adjust $\tau_{intra}$.

Next, we study the effects of the starting time of such interventions on epidemic spreading (Figure 5). Intuitively, early local interventions yield a higher proportion of susceptible individuals. Moreover, nonlinear fitting shows that early local interventions play a more effective role in virus transmission than early global interventions. Specifically, Figure 5a shows that no matter how late the global intervention, early local interventions always yield high fraction of susceptible individuals. On the other hand, the final susceptible individuals reach less than 80% even if later local interventions are imposed (Figure 5b). We then use similar non-linear form to fit the curve, finding that the average effect of local intervention intensity is a = 4.52 (Figure 5a), which is larger than global intervention intensity (a = 4.15, Figure 5b).

### 3.3. The Incubation Period and Interventions

The main determinant of whether a continuous outbreak of an infectious disease can be classified as an ongoing epidemic is the rate of transmission of the disease. The incubation period $E_{t}$ of the disease is closely related to the transmission speed of the disease, which is an important basis for the prevention of infectious diseases. Here we ask, how can we intervene infectious diseases with different incubation periods effectively? To answer this question, we study the impact of different of incubation periods on epidemic spreading.

**Figure 6:**
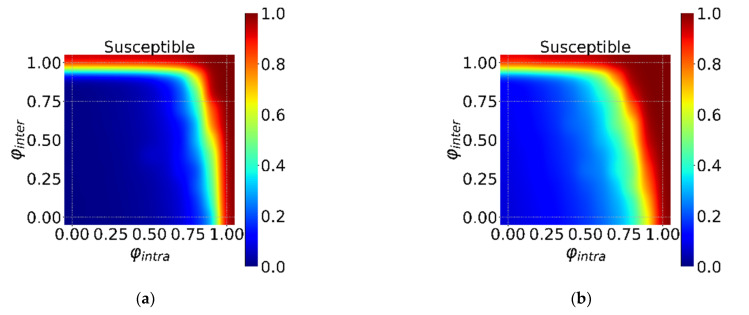

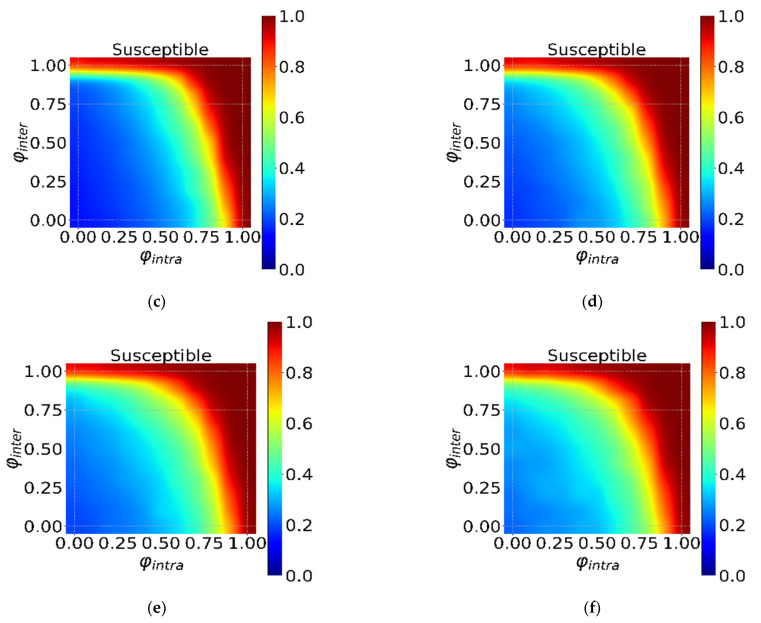
The interaction effect of the $\varphi_{intra}$ and $\varphi_{inter}$ under $\tau_{intra}=\tau_{inter}=0$. The horizontal axis is $\varphi_{intra}$, and the vertical axis is $\varphi_{inter}$ in (**a**–**f**). Different colors in the heatmap indicate different *S*. The ratio from dark red to dark blue transitions from 1 to 0 in turn. (**a**–**f**) show the different incubation periods $E_{t}$ ($E_{t}$ = 0, 5, 10, 15, 20, 25).

Figure 6 shows the effect of intervention intensity φ on epidemic spreading. Overall, the longer the incubation period, the easier it is for intervention to suppress the spread of epidemics. This shows that for epidemics with a short incubation period, only strong intervention can inhibit the spread of the virus, otherwise a global pandemic will break out. Moreover, there is an obvious transition line (*S* ≈ 0.8), below which infectious diseases erupt out of control. And as the incubation period grows, the dividing line gradually moves to the lower left, which also shows that the intensity that can suppress the spread of epidemics is inversely proportional to the length of the incubation period. Finally, strong intervention affects economic development. When resources are limited, different interventions based on the characteristics of infectious diseases can ensure the Pareto optimality to the maximum.

**Figure 7:**
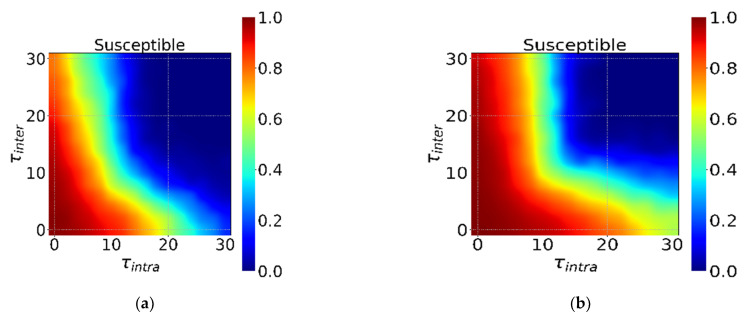

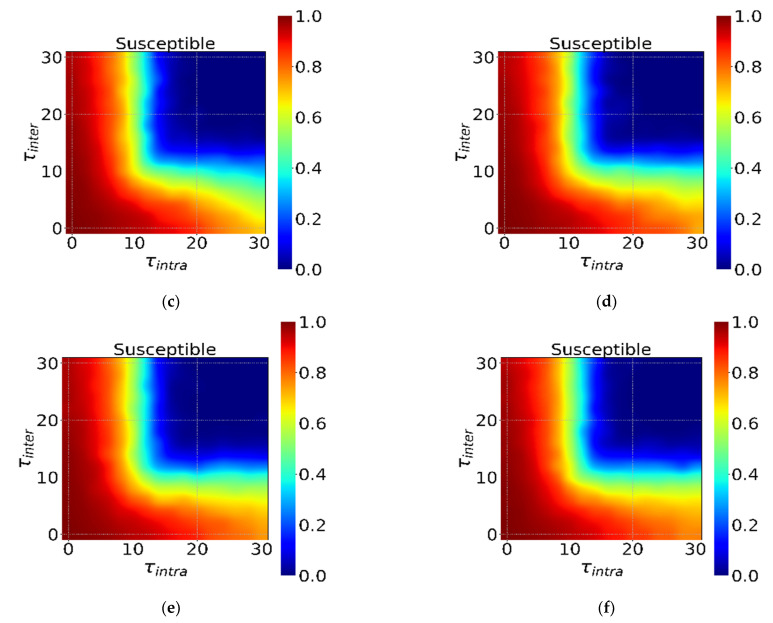
The interaction effect of the $\tau_{intra}$ and $\tau_{inter}$ under $\varphi_{intra}=\varphi_{inter}=0.9$. The horizontal axis is $\tau_{intra}$, and the vertical axis is $\tau_{inter}$ in (**a**–**f**). Different colors in the heatmap indicate different *S*. The ratio from dark red to dark blue transitions from 1 to 0 in turn. (**a**–**f**) show the different incubation periods $E_{t}$ ($E_{t}$ = 0, 5, 10, 15, 20, 25).

Figure 7 shows the impact of the start time of intervention τ during different incubation periods on the spread of the epidemic. First, we find similar conclusions as above, that is, the shorter the incubation period, the more it is necessary to start intervention as soon as possible. In addition, Figure 7 also has a similar transition line (*S ≈* 0.8) and a clear and stable inflection point, that is, the effect of intervention rapidly deteriorates when $\varphi_{intra}$ and $\varphi_{inter}$ are about 8–10 days later, the intervention effect rapidly deteriorates. This shows that infectious diseases in different incubation periods have similar intervention date critical points, which are not easy to change with changes in incubation periods. Finally, rapid intervention requires huge resources to support. Timely intervention when resources are abundant can effectively curb the outbreak of infectious diseases. When resources are limited, there is a Pareto optimal solution according to the specific conditions of social resources, which can most economically and effectively suppress a full-scale outbreak of infectious diseases.

### 3.4. Initial Distribution of the Infected Individual

Anecdotal evidence shows pandemic often exhibits local outbreaks within a city or a community rather than random outbreaks. Does such initial infected distribution affect the results? Which intervention strategy is effective under different conditions? To answer these questions, we study the impact of the initial distributions of infected individuals on epidemic spreading. We set initial infected individual density to be ρ = 0.025 (I(0)≈50 in the LFR networks). The network initialization and experimental details are in the Supplementary Materials. The initial infected distribution is the random distribution and the regional distribution. The regional distribution refers to the distribution of the infected nodes in a same region while the random distribution means that the infected nodes are randomly distributed throughout the entire network.

**Figure 8:**
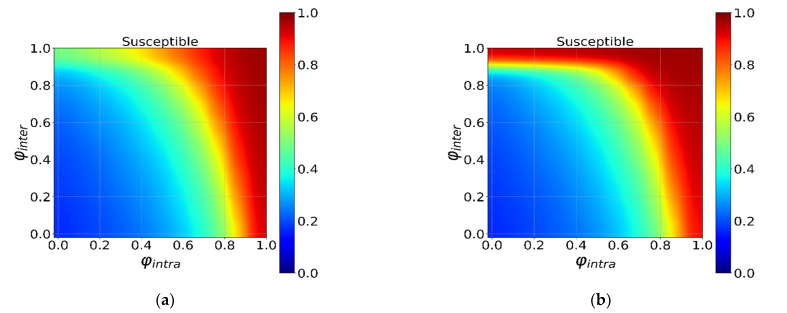
The interaction effect of the $\varphi_{intra}$ and $\varphi_{iner}$ under $\tau_{intra}=\tau_{inter}=0$. The horizontal axis is $\varphi_{intra}$, and the vertical axis is $\varphi_{iner}$ in (**a**,**b**). Different colors in the heatmap indicate different *S*. The ratio from dark red to dark blue transitions from 1 to 0 in turn. (**a**) The random distribution. (**b**) The regional distribution.

**Figure 9:**
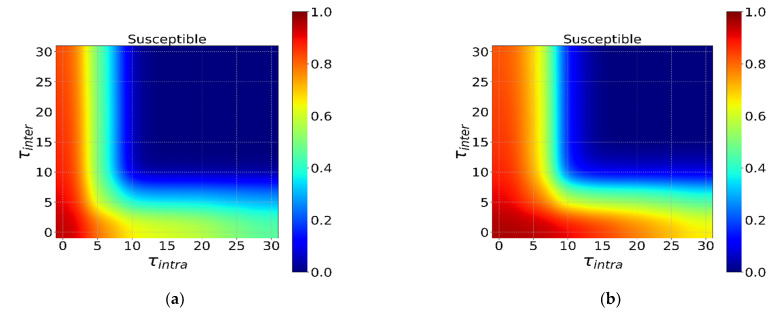
The interaction effect of the $\tau_{intra}$ and $\tau_{inter}$ under $\varphi_{intra}=\varphi_{inter}=0.9$. The horizontal axis is $\tau_{intra}$, and the vertical axis is $\tau_{inter}$ in (**a**,**b**). Different colors in the heatmap indicate different *S*. The ratio from dark red to dark blue transitions from 1 to 0 in turn. (**a**) The random distribution. (**b**) The regional distribution.

In this part, we study the effect of intervention intensity φ and starting time τ on virus transmission under different initial distributions. Figure 8 shows the effect of travel restriction intensity φ. Overall, strong local intervention intensity yields a high proportion of susceptible individuals (red area on the right side of Figure 8), indicating that local intervention strategy is effective for both distributions. While a global intervention strategy is more effective in suppressing the transmission of the epidemic under the regional distribution. To further discuss the effects of global and local interventions under different scenarios, we performed a multiple linear regression, where the dependent variable is the fraction of susceptible individuals and independent variables are the intensity of local and global intervention strategies. We find that the coefficients of local and global intervention strategy of random distribution are respectively 0.7063 and 0.3196, and of regional distribution are 0.6639 and 0.4281. The local intervention strategy shows consistent higher coefficient, which suggests that local intervention strategy in general has a more important role than global intervention strategy in both distributions. Moreover, global intervention strategy is necessary when the initial infected individuals are in the same region, as high global intervention intensity effectively inhibits the spread of the virus. Regression results also suggest similar conclusion as the coefficient of global intervention strategy increases substantially. Figure 9 shows the effect of intervention starting time on the epidemic spreading under different initial distributions, and we find similar conclusions. Specifically, regression coefficients of local and global intervention strategy under the random initial condition are −0.0220 and −0.0133, respectively. The coefficients under local initial condition are −0.0239 and −0.0183.

## 4. Conclusions

This paper developed a new model that contains “quarantine status” to study the effect of global and local interventions on epidemic spreading. Our findings that such interventions can effectively reduce the transmission of pandemic are consistent with previous studies [28,29,30,31,32,33,34,35,36]. What’s more, we studied the relationship between different incubation periods and intervention measures, finding that early implementation of rigorous intervention significantly reduced the scale of the epidemic, and there is a Pareto optimal in the intensity and time of intervention when resources were limited. Moreover, we systematically compare the efficiency of global and local interventions, finding that intense and earlier implementation of local interventions could notably reduce the magnitude of the outbreak. Finally, under the regional initial condition that is similar to real-world cases, combining local and global interventions reduces the magnitude and delays the peak of the infected individuals, but at the same time failure to implement such strategies would have accelerated the spread of the virus.

Our results suggest several key points. First, our simulation results support and validate the idea that global and local interventions such as travel restrictions and school/workplace closure have significantly reduced the epidemic’s spreading. Second, the local and global interventions have different roles. Global interventions have substantially reduced population movements between different regions/countries (for example, countries have adopted measures for ships/flights/trains, and people’s entry), while local interventions have reduced contacts between people in the same region (for example, lockdowns, prohibit large-scale gatherings, close entertainment venues, advise people to reduce going out, etc.). By comparing these two strategies, we find local interventions are systematically more important than global ones. What’s more, early implementation of rigorous intervention significantly reduced the scale of the epidemic under ideal conditions. However, when resources are limited, scientific and reasonable interventions based on the characteristics of infectious diseases according to the specific conditions of social resources, which can most economically and effectively suppress a full-scale outbreak of infectious diseases. Finally, our simulation results show that when the virus initially presented a random distribution, although global intervention strategies had a certain effect on reducing the virus in the early stages of transmission, local interventions were more effective in comparison. Although global intervention strategies also played an equally important role, local intervention strategies were more effective in suppressing the spread of the epidemic under regional distribution. This further demonstrates that in the early stage of the epidemic, it is important to timely implement inter-regional travel restriction such as city lockdown, border controls, etc. During the spread of the epidemic, the importance of inter-regional travel restriction, such as home isolation, social distance, etc., is more important for epidemic prevention and control.

Our work is not without limitations. First, we do not consider social and economic cost of such interventions in the model. In fact, travel restrictions carry a significant social/economic cost, which limits the duration of restrictions and affects the actual implementation. Second, our model parameters are static, which may not be true in real-world cases. For example, the cure rate (mortality rate) of infected individuals may increase (decrease) with time, but in this model, it is set as a constant.

Finally, our paper is a guideline for such global and local interventions. The effectiveness of different interventions varied, while these interventions that were used to contain the outbreak in various countries appear to be effective. According to many situations, deployment and adjustment of interventions are made to maximize the benefits of these interventions. Our work on combining local and global interventions improves our understanding of the impact of global and local interventions on pandemic, and will provide a reference for global response.

## Figures and Tables

**Table 1:** Parameters of the LFR benchmark network.

Parameter	Meaning	Value
*N*	Number of nodes in the created graph	2000
*k*	Desired average degree of nodes in the created graph	15
*maxk*	Maximum degree of nodes in the created graph	50
*minc*	Minimum size of communities in the graph	40
*maxc*	Maximum size of communities in the graph	70
*μ*	Fraction of intra-regional edges incident to each node	0.18

## Data Availability

The data supporting our results come from our designed procedure. The procedure code and the results are included within the Supplementary Material files.

## Supplementary Materials

The following are available online at https://www.mdpi.com/article/10.3390/ijerph182312627/s1. A concise description of each supplementary material file is listed in Table S1. The folder “code” contains the source code for the model proposed in the paper. The folder “results of data” contains all the results of running the program. Specifically, it includes all the experimental results: comparing non-pharmaceutical interventions and null model, exploring global and local interventions in terms of intensity and timing, analyzing different initial distributions.
