# Supplementary material for: The Effect of Local and Global Interventions on Epidemic Spreading
Source: Int J Environ Res Public Health. 2021 Nov 30;18(23):12627. doi: 10.3390/ijerph182312627 (PMC8657414; doi:10.3390/ijerph182312627)
Supplement: Supplementary file 1 [file ijerph-18-12627-s001.zip › Supplementary Materials/Supplementary Materials.pdf]

Supplementary Materials

1、

A concise description of each supplementary material file is listed in Table S1 below.

The folder “code” contains the source code of the multi-regional-SQEIR travel restriction model in the paper.

The folder “results of data” contains all the results of running the program. Specifically, it includes experimental results from running on regular networks, random networks, and benchmark networks, as well as all the topological graphs from running the regular networks via three algorithms.

Table S1. The concise description of each supplementary material file

<table>
<tr><th colspan="2">folder</th><th>file</th><th>description</th></tr>
<tr><td colspan="2" rowspan="5">code</td><td>main.py</td><td>Source code of the multi-regional-SQEIR travel restriction model.</td></tr>
<tr><td>sTrans.py</td><td>Source code of the state transition of susceptible individual.</td></tr>
<tr><td>oTrans.py</td><td>Source code of the state transition other individuals.</td></tr>
<tr><td>LFR_to_mine.py</td><td>Preprocess the LFR network to obtain the required data.</td></tr>
<tr><td>LFR.exe</td><td>The program that generates the LFR network.</td></tr>
<tr><td rowspan="5">results of data</td><td rowspan="4">1</td><td>I_Both.json</td><td>Experimental results of the infected under multi-regional-SQEIR model.</td></tr>
<tr><td>I_None.json</td><td>Experimental results of the infected under Null model.</td></tr>
<tr><td>S_Both.json</td><td>Experimental results of the susceptible under multi-regional-SQEIR model.</td></tr>
<tr><td>S_None.json</td><td>Experimental results of the susceptible under Null model.</td></tr>
<tr><td>2</td><td>the intensity_S.json</td><td>Experimental results of the susceptible individual with different interventions.</td></tr>
</table>

		the starting time_S.json	Experimental results of the susceptible individual with different starting time of interventions.
	3	the intensity_S.json	Experimental results of the susceptible individual with different interventions under the different incubation periods.
		the starting time_S.json	Experimental results of the susceptible individual with different starting time of interventions under incubation periods.
	4	random_intensity_S	Experimental results of the susceptible individual with different interventions under the random distribution.
		random_starting time_S	Experimental results of the susceptible individual with different starting time of interventions under the random distribution.
		regional_intensity_S	Experimental results of the susceptible individual with different interventions under the regional distribution.
		regional_starting time_S	Experimental results of the susceptible individual with different starting time of interventions under the regional distribution.

2、We simulate the model on the LFR network [51] to study the impact of travel restriction strategy on epidemic spreading. The main parameters of LFR benchmark network are as shown in Table 1 in the main text.

The specific parameters of this experiment are shown in Table S2.

In section 3.1, 3.2 and 3.3, we generate an initial network according to the LFR model. The initial fraction of affected individuals is ρ = 0.001, which are randomly assigned to each node in the network. We made 10 different initial assignments under the same LFR network, and each initial assignment carried out 50 complete iterative processes (the results were steady-state). Finally, the average of 500 results is taken as the final experimental result.

In section 3.4, the initial fraction of affected individuals is ρ = 0.025. We assign the state to the node according to the ρ and initial distributions. For the random distribution,

the affected individuals are randomly assigned to each node in the network. While for the regional distribution, we select a region (i.e., community) with more than 50 nodes randomly and assign affected individuals only within this community. We make 5 different initial node assignments under the same initial network, and then conduct 50 complete iterative processes under each different assignment (the results are steady-state). Finally, the average of 250 results is taken as the final experimental result.

The results are not sensitive to the parameter.

Table S2. Parameters and value of the experiment

Parameters	β	β_1	σ	q	γ	α
**Value**	0.8	0.3	0.33	0.0005	0.3	0.05

In order to established the epidemic model in the complex network, the status of nodes in the network will be initialized at first. At the beginning, the nodes in the network only have the susceptible and the exposed, and the number of the I status nodes is determined by the parameter ρ. Secondly, the node v_i in the network is updated in turn ($\{v_i\} \in V \to \{1,2,3,\dots,N\}$), and the updating rules are shown in Eq. 1. When the status of all nodes has completed, which is called an iteration. We set the maximum number of iterations $I_{max} = 1000$. When the total number of all status nodes is no longer updated for 50 consecutive iterations, it will jump out of the loop. The pseudo code of the main model is as follows:

The multi-regional-SQEIR travel restriction Model

1. Input: The initial network: $G(V,E)$; The initial susceptible and exposed distribution; The 6 status ;The series of parameter about model: $\beta, \beta_1\ q, \sigma, \gamma, \alpha$; The incubation period: E_t; The current quarantine period: $t_{qvi} = 0$; The parameters of travel restriction: τ_{intra}, τ_{inter}, φ_{intra}, φ_{inter}; The maximum of iterations: I_{max}.
2. While $t < I_{max}$;
3.   For v_i in (V);
4.   If v_i is the susceptible:
5.   If $t<\tau_{intra}$ and $t<\tau_{inter}$:
6.   Update the status of v_i according to v_i's neighbors under none travel restriction;
7.   Elif $t>\tau_{intra}$ and $t<\tau_{inter}$:
8.   Update the status of v_i according to v_i's neighbors under intra-travel restriction;
9.   Elif $t<\tau_{intra}$ and $t>\tau_{inter}$:

10. Update the status of v_i according to v_i's neighbors under inter-travel restriction;
11. Elif $t > \tau_{intra}$ and $t > \tau_{inter}$:
12. Update the status of v_i according to v_i's neighbors under both travel restrictions;
13. Elif v_i is the quarantine:
14. If $t_{qvi} < E_t$:
15. v_i might change to the I status with q,
or $t_{qvi} += 1$;
16. Else:
17. v_i might change to the S status;
18. Elif v_i is the exposed:
19. $t_{qvi} = 0$;
20. v_i might change to the I status with σ,
or v_i would stay the same with $(1-\sigma)$;
21. Elif v_i is the infective:
22. $t_{qvi} = 0$;
23. v_i might change to the R status with γ,
or v_i might change to the D status with α,
or v_i would stay the same with $(1-\alpha-\gamma)$;
24. Update the 6 status: $S(n), E(n), Q(n), I(n), R(n), D(n)$;
25. If all status nodes stay the same for 50 iterations:
26. Break;
27. Output: the 6 status.

To further illustrate the travel restriction strategy, the pseudo codes of the travel restriction strategy are as follows:

The travel restriction strategy.

1. Input: The initial network: $G(V,E)$; The initial susceptible and exposed distribution; The 8 status: ;The series of parameter about model: $\beta, \beta_1\ q, \sigma, \gamma, \alpha$; The parameters of travel restriction: $\tau_{intra}, \tau_{inter}$; The incubation period: E_i; The current quarantine period: $t_{qvi} = 0$;
2. If $t < \tau_{intra}$ and $t < \tau_{inter}$, means there is none travel restriction:
3. For v_j in (neighbors of v_i):
4. If v_i is the susceptible:
5. v_i might change to the E status with β_1,
6. Else:
7. continue;
8. Elif $t > \tau_{intra}$ and $t < \tau_{inter}$, means there is intra-travel restriction:
9. If v_i and v_j are in the same regional:
10. If v_j is the Infective:
11. v_i might change to the E status with $\beta_1(1-\varphi_{intra})$,
12. or v_i might change to the S status with $\beta_1\varphi_{intra}$,
13. or v_i would stay the same;

14. Else v_i and v_j are in the different regionals:
15. If v_j is the Infective:
16. v_i might change to the *E* status with β_1,
17. or v_i might change to the *S* status with β_1,
18. or v_i would stay the same;
19. Elif $t<\tau_{intra}$ and $t>\tau_{inter}$, means there is inter-travel restriction:
20. If v_i and v_j are in the same regional:
21. If v_j is the Infective:
22. v_i might change to the *E* status with β_1,
23. or v_i might change to the *S* status with β_1,
24. or v_i would stay the same;
25. Else v_i and v_j are in the different regionals:
26. If v_j is the Infective:
27. v_i might change to the *E* status with $\beta_1(1-\varphi_{inter})$,
28. or v_i might change to the *S* status with $\beta_1\varphi_{inter}$,
29. or v_i would stay the same;
30. Elif $t>\tau_{intra}$ and $t>\tau_{inter}$, means there is both travel restrictions:
31. If v_i and v_j are in the same regional:
32. If v_j is the Infective:
33. v_i might change to the *E* status with $\beta_1(1-\varphi_{intra})$,
34. or v_i might change to the *S* status with $\beta_1\varphi_{intra}$,
35. or v_i would stay the same;
36. Else v_i and v_j are in the different regionals:
37. If v_j is the Infective:
38. v_i might change to the *E* status with $\beta_1(1-\varphi_{inter})$,
or v_i might change to the *S* status with $\beta_1\varphi_{inter}$,
or v_i would stay the same;
39. Output: the status of v_i.
